# Understanding the Two-Dimensional Mixing Behavior of 1-Naphthalenethiol and Octanethiol

**DOI:** 10.1021/acs.jpcc.2c08822

**Published:** 2023-03-24

**Authors:** Jack Sette-Ducati, Ryan Donnelly, Allison J. Molski, Emma R. Robinson, Emma K. Canning, Daniel J. Williams, Elizabeth C. Landis, L. Gaby Avila-Bront

**Affiliations:** Department of Chemistry, College of the Holy Cross 1 College St., Worcester, Massachusetts 01610, United States

## Abstract

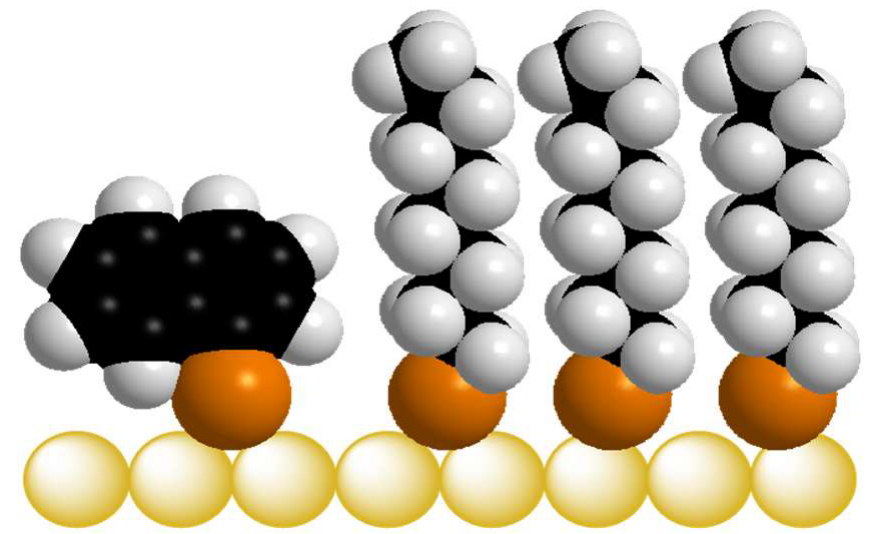

A two-dimensional (2D) mixture in the form of a self-assembled monolayer composed of two distinct organothiol compounds was created by sequentially depositing 1-naphthalenethiol (1NT) and octanethiol (OT) on a gold surface. By varying the sequence of deposition, two mixed surface systems were created. The surface structure of the resulting mixed monolayer was characterized with Scanning Tunneling Microscopy (STM) and showed surface disorder across all investigated domains. Elemental analysis was carried out with X-ray Photoelectron Spectroscopy (XPS) and indicated that the 1NT monolayer was prone to significant oxidation. Reductive desorption (RD) was used to characterize the binding strength and electrochemical environments of the molecular components in the mixture, and confirmed disordered molecular layers. Due to the presence of oxidized species in the 1NT monolayer, 1NT was displaced by OT resulting in a novel surface structure composed of either OT or 1NT. Monolayers of OT that were exposed to a solution of 1NT resulted in disordered surface structures with a significant amount of gold vacancy islands. To date, there is no experimental phase diagram explaining the chemical behavior of two-dimensional mixtures. This study addresses the need for an experimental understanding of the phase behavior of mixed organothiol self-assembled monolayers (SAMs).

## 1 Introduction

Searching the pre-existing literature for the term “self-assembled monolayer” (SAM) reveals an exponential growth in the study of these systems starting in the late 1980s. At that time, foundational studies reported the stability and robust capabilities of SAMs of organothiol compounds on curved or planar coinage metal surfaces.^1−8^ It has since been shown that the structure and functionality of a SAM can be tailored based on the chemical identity of the monolayer components. SAM-modified surfaces have been applied in fields ranging from tribology to nanotechnology to biology.^9−18^ These surface systems have exciting two-dimensional phase behavior that remains an emerging phenomenon.

Self-assembly is the process by which compounds spontaneously arrange themselves on a surface to maximize their van der Waals interactions.^5,6,19−22^ Single-component SAMs of aliphatic organothiol compounds on the surface of gold are the most extensively characterized system.^1,2,4,7,16,23−26^ Regardless of whether the SAMs are deposited from solution or the vapor phase, the formation mechanism is a two-step process: the first step is the chemisorption of the compounds to gold adatoms,^23^ and the second step is the formation of an energetically stable array via surface diffusion.^6,7,27^ During chemisorption, the sulfur moieties remove gold atoms from the surface, leaving behind islands of vacancies (*etch pits*).^23,28^ An approximate equilibrium structure is formed via surface diffusion of the compounds bonded to gold adatoms. Groups of the compounds coalesce into islands (*domains*) that fill in the monolayer. As the monolayer grows, these domains encounter one another and form fault lines (*domain boundaries*) if they are not structurally aligned.^24,28,29^ As the packing density of the monolayer increases, a phase transition occurs and the molecules transition from several lying down phases to a standing up, “crystalline phase” of hexagonally packed arrays.^1,28^ SAMs of aromatic thiols are considerably different from their aliphatic counterparts, and do not regularly form ordered arrays.^27,30−41^ Despite their disordered surface structures, their applications as molecular rectifiers make them highly attractive systems.^39,42,43^ SAMs formed from aromatic thiols have also found applications in nanotechnology due to their interesting electronic properties.^5,39^ SAMs formed from aromatic organothiol compounds that have been characterized with STM are marked by large domains of bright protrusions and an absence of gold vacancy islands.^37,44−47^

Mixed SAMs have gained traction as surfaces with preformed patterns that are not restricted to the size limitations present in lithography.^48−57^ These systems have shown the potential to act as templates for supramolecular layers,^53^ modifiers of a surface’s wettability,^48^ or electron transfer properties.^55^ To date, the only mixed SAMs to have been created have been formed by the adsorption of two compounds on a surface and are termed *binary monolayers*.^44,45,58−62^ The most studied binary SAMs are those of aliphatic thiols composed of different lengths or functional groups, with little work done on binary SAMs composed of aliphatic and aromatic thiols. Binary monolayers are created by either coadsorption^61,63−65^ (adsorption from a common solution) or sequential adsorption^44,45,59,62^ (adsorption of one component followed by a second). Monolayers formed via coadsorption are influenced by competitive adsorption as well as solubility effects, while sequentially adsorbed binary SAMs highlight the role of defects in the existing monolayer. Modeling the phase behavior of binary SAMs with either Monte Carlo (MC) or Density Functional Theory (DFT) indicates that phase separation occurs when the intermolecular interaction energy exceeds the intramolecular interaction energy and that phase transitions are dependent on molecule length and temperature.^66−74^ Incorporating multiple compounds within a single SAM creates a complex surface system. The chemical identity of the compounds, their bulkiness, the concentrations of the deposition solutions, annealing temperatures, and the method of deposition all affect the final monolayer structure composed of homogeneous or phase-separated domains.^1,56,57,75,76^

Reductive desorption (RD) has been demonstrated as an accurate and precise analytical technique used to investigate the stability, structure, degree of order, and electrochemical environment of SAMs. RD can be especially useful in probing binary SAMs, so long as the peak potentials of the compounds are distinguishable.^77^ By applying a negative bias to a surface that has been modified with a single-component or binary SAM, the adsorbate is reduced and subsequently desorbs from the surface. It is widely accepted that the one-electron reduction reaction leading to the desorption of organothiol SAMs from gold surfaces is^78−80^
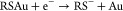
1Organothiol SAMs generally desorb at negative peak potentials. The shape of the reductive peaks in the cyclic voltammograms is indicative of the desorption process for a compound. Sharp peaks indicate that desorption occurred readily near the peak potential and that the desorbing compounds were present in the same electrochemical environment, signifying an ordered domain.^81^ Broad peaks point to a complicated desorption process with desorbing compounds present in a wide range of environments. There is evidence that these complicated desorption processes are the result of inhomogeneity of the surface,^82^ and nonuniform structures of the adsorbate layer.^83,84^ Reductive desorption studies of alkanethiolate monolayers are the most widely published most likely because the surface structures of these SAMs are highly ordered and lead to well-defined reductive peaks.^79−81,85^ On the other hand, the tendency of arylthiols to produce disordered monolayers leads to broader spectra that are harder to interpret. Nevertheless, RD studies on arylthiol are systematically being carried out and reveal a system of rich chemical interactions.^83−90^

In this present work, we have created and analyzed the binary SAM formed from the sequential deposition of 1-naphthalenethiol (1NT) and octanethiol (OT) on a gold surface in ambient conditions. OT was selected as the aliphatic component of the binary SAM due to its well-characterized surface behavior.^6^ Binary monolayers present unique experimental challenges. For example, the difference in tunneling currents between dissimilar compounds makes characterization via STM (a staple of surface science due to its ability to molecularly resolve surface structures noninvasively) difficult. While there are slight differences between carbon signals from aliphatic and aromatic compounds, these differences are not always pronounced enough. As a result, characterization via XPS is further complicated. Our study represents the first characterization of a binary monolayer composed of these two components. Resolution of the structure of the binary SAM is carried out with STM, and elemental analysis is characterized by XPS. RD is used to understand the electrochemical stability of the binary SAM. Due to the complex nature of these systems, fundamental studies creating and analyzing the structure of binary SAMs are of particular interest to the surface science community. As aromatic organothiols have been established as candidates for nanotechnological applications, such as organic molecular wires, understanding their behavior in ambient and nonpristine conditions is of utmost importance.^18,39^

## 2 Materials and Methods

Samples were prepared using Au(111)-on-mica substrates (Keysight Technologies), which were flame-annealed with a hydrogen flame prior to use. Exposure to light was minimized during all deposition processes by maintaining the samples under a dark cover to minimize photooxidation.

OT monolayers were prepared by placing the bare, flame-annealed Au(111) surface in a 20 mL glass scintillation vial. A separate vial containing 5 μL of the OT liquid (Sigma-Aldrich, 98.5%) was placed adjacent to the surface to ensure that the surface did not come into contact with the liquid, to avoid oversaturation of the surface and the formation of multilayers. The 20 mL vial was then capped, covered with aluminum foil, and placed in an oven at 70°C for 18 h, during which time the OT was vapor deposited onto the Au(111) surface. This method was adapted from the method described by Deering et al.^28^

1NT monolayers were prepared by immersing the bare, flame-annealed gold surface in a 1 mM ethanolic solution of 1NT (Sigma-Aldrich, 97%) for 24 h at 60 °C. These samples were cooled down for 1 h in the dark, vigorously rinsed for 3 min with methanol, and dried under a nitrogen stream. Several rinsing solvents were tested in order to reduce the likelihood of physisorbed compounds interfering with STM measurements. Samples were rinsed with benzene, pyridine, ethanol, and methanol. It was established that samples rinsed with methanol or ethanol resulted in the clearest STM images. Vapor deposition of 1NT was also attempted but resulted in very low coverage of the gold surface.

Binary monolayers were prepared by putting together the individual deposition methods while varying which compound was deposited first. We will refer to surfaces in which OT was adsorbed first and then immersed in a solution of 1NT as OT/1NT. OT/1NT monolayers were prepared by vapor-depositing OT at 70 °C for 18 h. Once the OT had been deposited, the surface was immersed into a 1 mM 1NT solution for 24 h at 60 °C. We will refer to surfaces in which 1NT was adsorbed first and then exposed to OT vapor as 1NT/OT. 1NT/OT monolayers were prepared by immersing the bare, flame-annealed Au(111) surface in a 1 mM 1NT solution for 24 h at 60 °C. Following this immersion, the surfaces were exposed to OT vapor for 18 h at 70 °C. All binary samples were rinsed with ethanol after exposure to OT and dried with N_2 (g)_.

Images were obtained at room temperature and under atmospheric conditions using a scanning tunneling microscope (Keysight/Agilent Technologies 5100 Series STM and AFM) in constant current mode equipped with hand-cut 0.25 mm Pt80/Ir20 wire (Goodfellow Cambridge Limited) and operated under PicoView 1.14.4 software (Keysight/Agilent Technologies). Instrumental parameters were set with a constant tunneling current of 30–80 pA and a sample bias of 0.3–0.8 V. All samples were imaged immediately following the last deposition step to avoid sample degradation. Images were analyzed and processed using WSxM 5.0 software.

Elemental analyses of the single-component and binary monolayers were conducted by X-ray photoelectron spectroscopy (XPS). Samples were removed from their final deposition vials and moved to dark containers 1–2 h before being transferred to the XPS chamber. XPS measurements were performed using a monochromatic Al–Kα X-ray source (Thermo Scientific, K-Alpha+ X-ray Photoelectron Spectrometer System). Spectra were acquired with 50 eV pass energy and a step size of 0.1 eV. XPS peaks were fit using the Thermo Avantage v.5.9925 software.

Reductive desorption measurements were performed by attaching the gold electrodes to glass slides with epoxy for stabilization, to which a top electrical contact was established by placing double-sided conductive tape on the sample and coating it with epoxy so only the gold surface was exposed to electrolyte solution. The sample was placed in an electrochemical cell with 0.5 M KOH in milli-Q water with a Ag/AgCl reference electrode and a Pt counter electrode. Cyclic voltammetry was performed on an FRA2 μAutolab Type III potentiostat from 0 V to −1.4 V with a scan rate of 0.1 V/s. Measurements were collected in triplicate, and uncertainties represent the standard deviation of the measured values.

## 3 Results and Discussion

**Figure 1:**
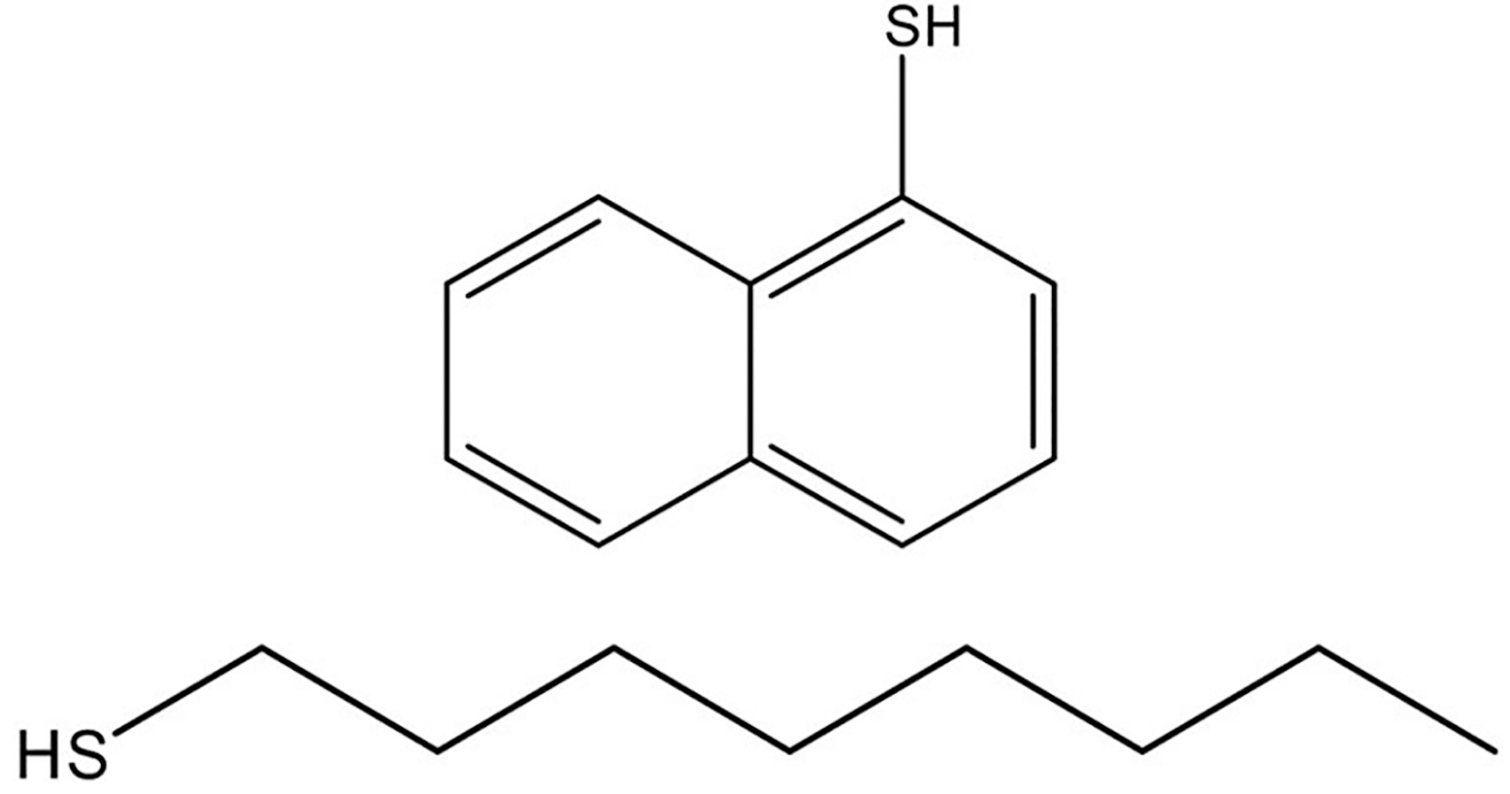
Chemical structures octanethiol (OT) and 1-naphthalenethiol (1NT), the compounds used in this study.

Figure 1 shows the chemical structures of the compounds investigated in this study. OT has a length of 8.06 Å, and 1NT has a length of 6.8 Å from the outer edge of one benzene ring to the outer edge of its neighboring benzene ring. These measurements were obtained using the software Avogadro.^92^

### 3.1 STM Images of Single-component Monolayers of OT and 1NT

**Figure 2:**
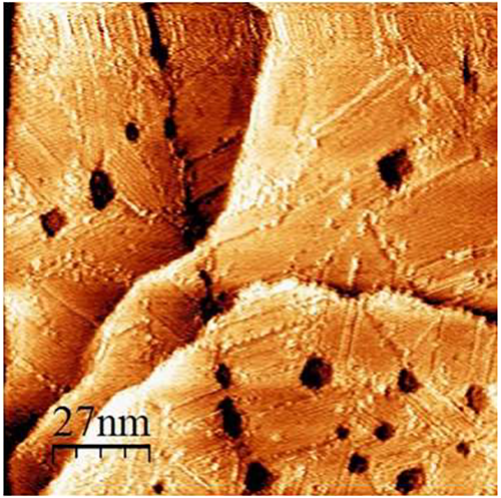
Representative STM image of single-component OT on Au(111) vapor deposited at 70 °C.

**Figure 3:**
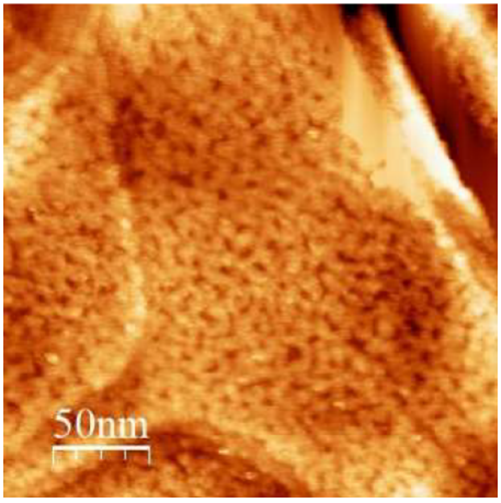
Representative STM image of single-component 1NT formed by a 24 h immersion at 60 °C in a 1 mM ethanolic solution, rinsed with methanol, cooled down for 1 h prior to imaging, rinsed for 3 min with methanol, and dried with N_2_.

Figures 2 and 3 are representative STM images of the single-component monolayers. The SAM structure of OT is well-known in the literature.

Figure 3 is a representative STM image of a 1NT SAM on Au(111). This image is, to the best of our knowledge, the only STM images of a 1NT SAM on the surface of gold in ambient conditions. Overall, STM data of a monolayer of 1NT in ambient conditions reveal a disordered surface with features that resemble the porous structure of sea coral. These resolved surface structures are assigned to the adsorbed compound. In Figure 3, an 160 nm × 160 nm area is presented in this image, which contains one main terrace that is covered by a disordered monolayer of 1NT that traverses step edges to form complete coverage of the area. Step edges appear to be scalloped, indicating the adsorption of the monolayer in those areas. Although the molecular structure of 1NT shows the possibility of an ordered monolayer being stabilized by parallel-displaced pi–pi interactions, the reality of the monolayer is quite different. Other aromatic organothiols have been shown to form disordered SAMs characterized by well-defined bright protrusions on the surface.^37,44^ The surface structure of 1NT does not exhibit these features. Rather, the features of a 1NT SAM bleed into one another and are not clearly defined in any way.

**Figure 4:**
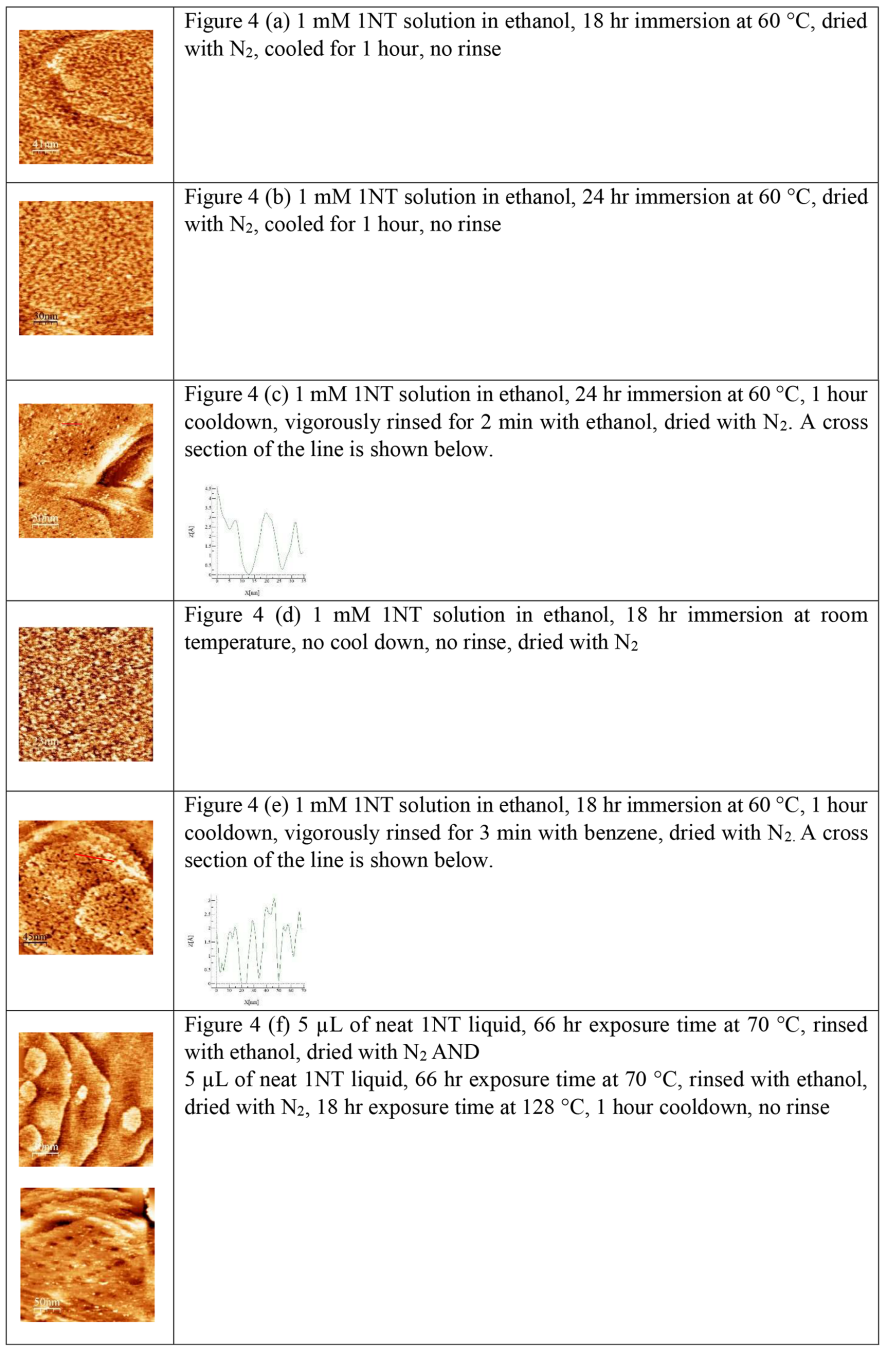
Representative STM images with various deposition conditions are shown. This figure represents the only STM images of 1NT on Au(111) in the literature. While the images show drastic differences between solution-deposited and vapor-deposited 1NT monolayers, the differences between solution-deposited samples with varying rinsing, cooldown periods, and deposition times are seemingly not substantial. During the “cooldown” periods, samples were allowed to rest at room temperature in a container free from any sources of light.

As our study represents the only known investigation of a 1NT SAM in ambient conditions, the deposition variables of time, phase, temperature, and rinsing solvent were explored. Previous studies have demonstrated the important effects that these variables can have on the resulting surface structures of a monolayer.^92,93^Figure 4 summarizes the representative images from each of the attempted deposition methods. There are little to no differences in the physical appearances of solution deposited samples that varied in deposition time, temperature, or rinsing solvent. Each of these samples resulted in disordered adsorbate layers. However, there were differences in the noise/blurriness of these samples during the scanning process. While scanning, samples that had been prepared with the deposition method detailed in the Methods section resulted in images that were the least noisy and the clearest. The largest differences were observed in samples in which 1NT was vapor deposited. Vapor-deposited adsorbate layers resulted in surfaces that resembled bare gold, with no indication of the bright features commonly observed in solution-deposited samples. XPS confirmed submonolayer coverage for this deposition technique. An increase in the coverage of the surface by 1NT correlates with the emergence of these surface structures and informed our previously discussed designation.

### 3.2 STM Images of Binary Monolayers of OT/NT and 1NT/OT

#### 3.2.1 OT/1NT

**Figure 5:**
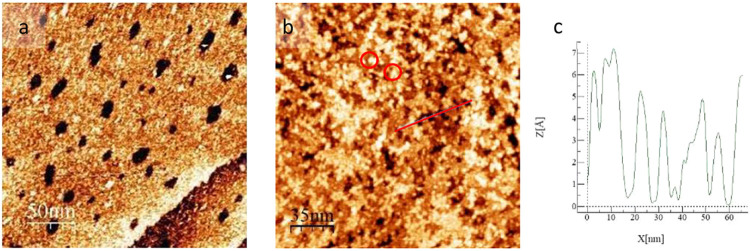
Representative STM images of OT monolayers immersed in ethanolic 1 mM 1NT solutions at 60 °C for 24 h. Surfaces were rinsed with methanol and dried under a Nitrogen stream before imaging. Panel (a) shows the presence of an OT monolayer by the presence of etch pits. These etch pits are surrounded by bright features. Panel (b) shows bright clusters completely covering the area. Panel (c) shows a cross section of the line highlighted in the (b).

Binary monolayers of OT and 1NT were created via sequential adsorption, as aforementioned. When existing OT monolayers were immersed in a 1 mM ethanolic solution of 1NT, the resulting surfaces were highly disordered. Two distinct types of disordered surfaces were observed. Figure 5 shows representative images of the observed surface structures. In panel (a), we observe a large terrace with a step edge separating it from a small terrace in the lower right-hand corner of the image. Both terraces are covered in etch pits. These etch pits indicate the presence of an alkanethiolate monolayer (OT in our case), as monolayers of aromatic organothiols do not typically result in a pitted gold surface. Completely covering this area are bright features with small consistency in size. This type of structure resembles degraded OT monolayers that have lost the crystallinity of the OT domains, and retain only the underlying gold surface that has been eaten away by thiol head groups. It is possible that these features are gold adatoms, but due to the variability in their size and shape, we are unable to discern the chemical nature of these features with STM.

Panel (b) of Figure 5 shows a representative image of the secondary type of surface structure observed in binary OT/1NT monolayers. In this image we observe the center of a terrace with no visible step edges present. Though there are dark areas present in this image, these regions do not appear to be etch pits. Rather, the presented area is covered in bright clusters and islands. In general, the islands cannot be measured as the features bleed into each other. When the features are distinguishable (circled in the Figure), they measure between 1.98 and 6.83 nm. These images verify that 1NT is adsorbing to the surface in a limited and disordered capacity.

#### 3.2.2 1NT/OT

**Figure 6:**
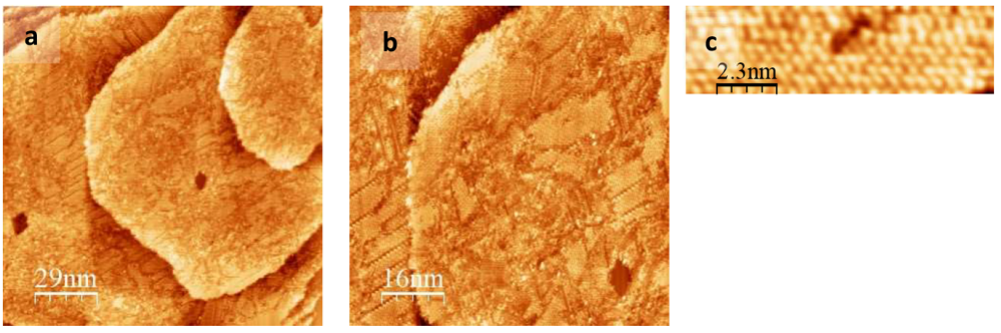
Representative STM images of binary 1NT/OT monolayers. This sample was prepared by immersing a flame-annealed gold in a 1 mM ethanolic solution of 1NT at 60 °C for 24 h. The sample was rinsed with methanol and dried under a Nitrogen stream before being exposed to OT vapor at 70 °C for 18 h. Prior to imaging, the sample was rinsed with ethanol and dried under a Nitrogen stream. Each panel is a zoom-in of the previous.

Exposing a 1NT SAM to OT vapor resulted in the replacement of 1NT by OT. The resulting observed surface structures greatly resembled single-component OT monolayers, albeit without as much global order as observed in a typical single-component OT SAM. Representative images of these surfaces are shown in Figure 6. A wide-scale area of 134 nm × 142 nm is shown in Panel (a) and demonstrates that while the OT is self-assembling into ordered domains, these domains do not traverse the entirety of the surface. In the image presented in Panel (a), we observe three main terraces, with patches of ordered domains within the terraces. Zooming into these domains reveals domains of hexagonally packed features with a spacing of 0.53 ± 0.06 nm. This spacing is consistent with the characteristic with OT on Au(111) surfaces. The spread in the average spacing is attributed to drift.

**Figure 7:**
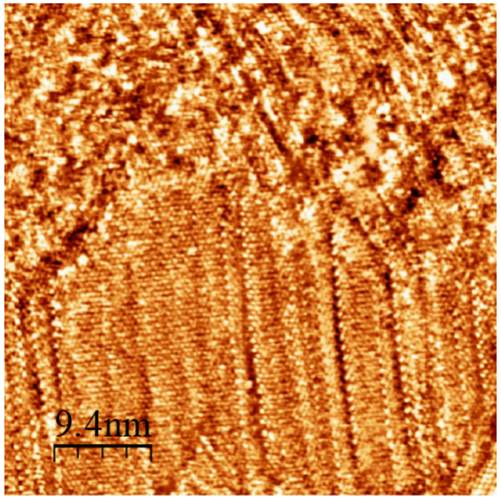
Representative STM images of binary 1NT/OT monolayers displaying the displacement of 1NT by OT. This sample was prepared by immersing a flame-annealed gold in a 1 mM ethanolic solution of 1NT at 60 °C for 24 h. The sample was then rinsed with ethanol and dried under a Nitrogen stream before being exposed to OT vapor at 70 °C for 18 h. Prior to imaging, the sample was rinsed with ethanol and dried under a Nitrogen stream.

Figure 7 shows a particularly interesting domain of OT. In this image, we observe a large domain with an angled boundary of 110°. The bright features in between the columns of domain boundaries have a spacing consistent with that of OT on Au(111). The bright features within the rows are spaced 0.9 ± 0.2 nm. Due to the fact that STM images only measure topographical and geometrical information, we cannot state the chemical nature of these bright features. It is likeliest that these features are OT molecules, but cannot say with certainty that they are not 1NT. We believe it is unlikely that they are 1NT because we have not seen any indication of molecular resolution of this compound.

### 3.3 XP Spectra

#### 3.3.1 S 2p Peak of Single-Component OT and 1NT SAMs

**Figure 8:**
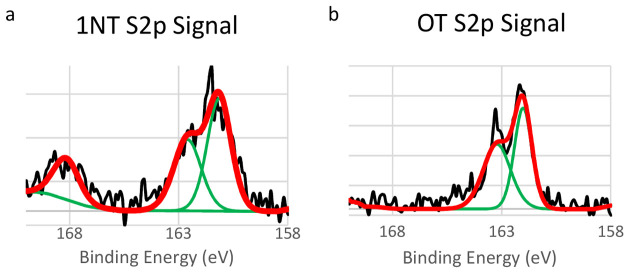
XP spectra of single-component 1NT and OT monolayers. The S 2p signal is shown. On the graphs, the raw data signal is shown in black, the curves used to fit the data are shown in green, and the final fit is shown in red.

XPS was used to conduct elemental analysis of single-component and binary SAMs. In Figure 8a, the XP spectrum of 1 mM 1NT single-component SAMs, which has not previously been reported in the literature, shows a single S 2p_3/2_/S 2p_1/2_ doublet peak centered at 162 eV. This peak is indicative of a sulfur–gold bond. The shape and position of this peak is consistent with previous reports in the literature of similar compounds.^87,95^ A small baseline signal is present in the region of 165–167 eV which indicates the presence of physisorbed organosulfur compounds on gold.^4,92−94^ What is most notable of these spectra is the sizable peak present at 168 eV. This peak is attributed to the presence of sulfonate species and indicates a highly oxidized monolayer.^25,95−99^Figure 8b shows the S 2p XP spectra of a single-component OT SAM. The spectrum shows a single S 2p_3/2_/S 2p_1/2_ doublet peak centered at 162 eV, which is indicative of a sulfur–gold bond. The shape and position of this peak are consistent with previous reports.^4^

It has been shown that alkanethiol monolayers oxidize via air-oxidation and/or photooxidation. The oxidative process, when not deliberately carried out, varies with the amount to which samples are exposed to ozone and light. However, aromatic monolayers have previously been shown to be more resistive to oxidation than their aliphatic counterparts.^100^ Indeed, previous studies in our lab conducted in the same space and using the sample experimental techniques have resulted in aromatic monolayers with little to no oxidized sulfur species.^44,45^ Our samples are prepared in ambient conditions with minimal exposure to light. XP spectra (not shown) of samples rinsed with toluene and ethanol all resulted in oxidized monolayers. Samples not rinsed and deposited from the vapor phase also resulted in oxidized monolayers. We conclude that single-component monolayers formed from 1NT are particularly susceptible to oxidation. We attribute this effect to the highly disordered nature of 1NT monolayers that allows for oxidative processes to occur. The absence of a peak resulting from oxidized sulfonate species in OT XP spectra confirms the fact that our experimental methods do not cause oxidation of the SAM, but rather the nature of the 1NT SAM itself.

#### 3.3.2 C1S Peak of Single-Component OT and 1NT SAMs

**Figure 9:**
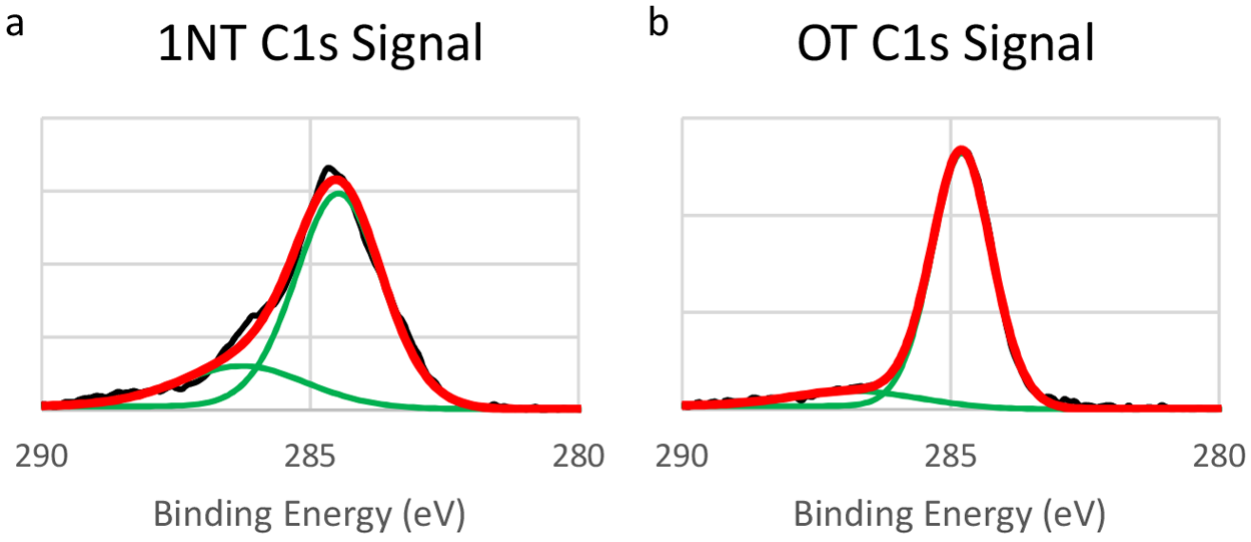
XP spectra of single-component 1NT and OT monolayers. The C 1s signal is shown. On the graphs, the raw data signal is shown in black, the curves used to fit the data are shown in green, and the final fit is shown in red.

The C 1s XP spectra of the single-component monolayers are shown in Figure 9. The C 1s spectra of 1NT deposited from the solution phase are shown in Figure 9a. The main contribution to the signal is positioned at 284.5 eV. This value is in good agreement with values reported for similar compounds previously studied in our group.^44,45^ For the most closely related compounds, Zareie et al. reported the C 1s signal for 2-naphthalenethiol (2NT) at 284.2 eV, while Frey et al. reported the C 1s signal at 284.0 eV for anthracenethiol.^94−96,100−102^ The position of the peak in this study, therefore, is higher than related compounds and indicates a less densely packed monolayer. This conclusion is confirmed by RD experiments discussed later in this section.

The main C 1s peak for the alkanethiol monolayers is shown in panel (b) and is centered at 284.8 eV. There is a slight difference in the C 1s signals between alkanethiolate SAMs deposited from the solution and those deposited from the vapor phase. Due to the lower coverage produced by deposition of the monolayer from the vapor phase, C 1s peaks for vapor-deposited samples appear at energies lower than the 285 eV peak observed for solution-deposited samples.^94−96^

#### 3.3.3 XP Spectra of Binary OT/1NT and 1NT/OT SAMs

**Figure 10:**
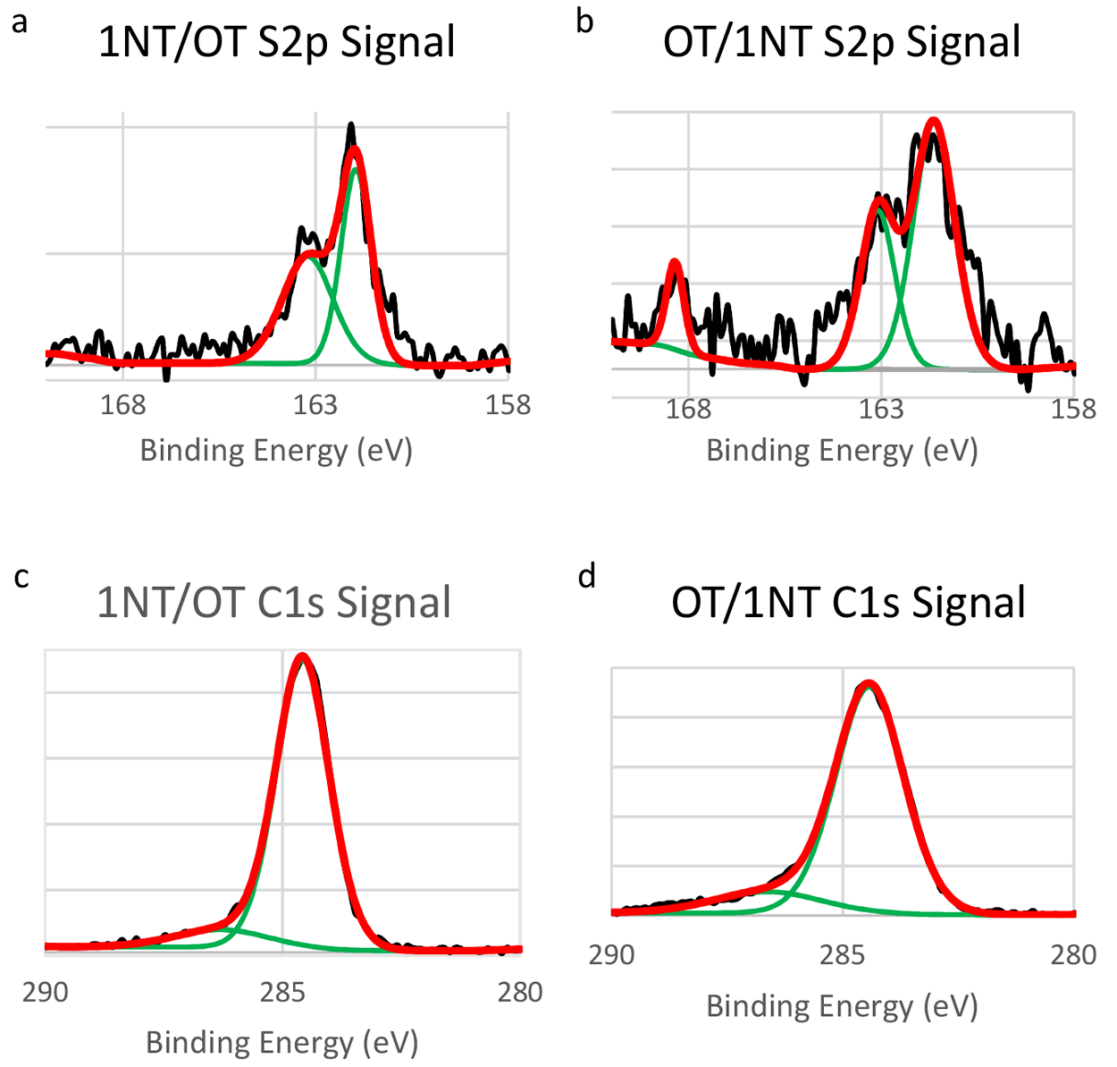
XP spectra of binary monolayers. The C 1s and S 2p signals are shown. On the graphs, the raw data signal is shown in black, the curves used to fit the data are shown in green, and the final fit is shown in red.

Binary monolayers produced spectra with similar binding energies for the C 1s peak, Figure 10a–b. In 1NT/OT binary SAMs, this peak was observed at 284.7 eV. When the order of deposition was reversed, OT/1NT binary SAMs showed the C 1s peak at 284.4 eV. We attribute the absence of a difference in the C 1s binding energies of these adsorbates as previously reported for aryl/alkanethiol systems to the submonolayer coverage of both 1NT and OT components.^94−96^

There is, however, a marked difference in the S 2p spectra for binary SAMs with opposing deposition sequence. As shown in Figure 10c, in binary SAMs where OT is deposited second (1NT/OT), the S 2p signal is composed only of a doublet centered at 162.0 eV. Conversely, the S 2p signal from OT/1NT monolayers has a doublet peak centered at 161.6 eV as well as a peak at 168.4 eV, signifying the presence of oxidized sulfur species in the form of sulfonates within the monolayer, Figure 10d. There is also a considerable amount of signal in the region between 163 and 165 eV, signifying physisorbed sulfur species. The oxidized sulfur peak is predominantly associated with 1NT monolayers, as seen in XP spectra of single component 1NT in Figure 8a. We conclude, then, that the presence of an oxidized sulfur peak indicates the increased presence of 1NT in the monolayer. We sought to quantify this statement by tracking the oxygen peak in our monolayers, but control experiments revealed that because our experiments are conducted in air, the background O_2_ levels on bare gold are particularly high and could not be used to quantify 1NT monolayer oxidation. However, the presence of this peak can indicate that there are enough available surface sites within the vapor-deposited OT monolayer to accommodate the adsorption of 1NT, and OT does not form a fully blocking monolayer.

## 4 RD CV of Single-Component and Binary SAMs

**Figure 11:**
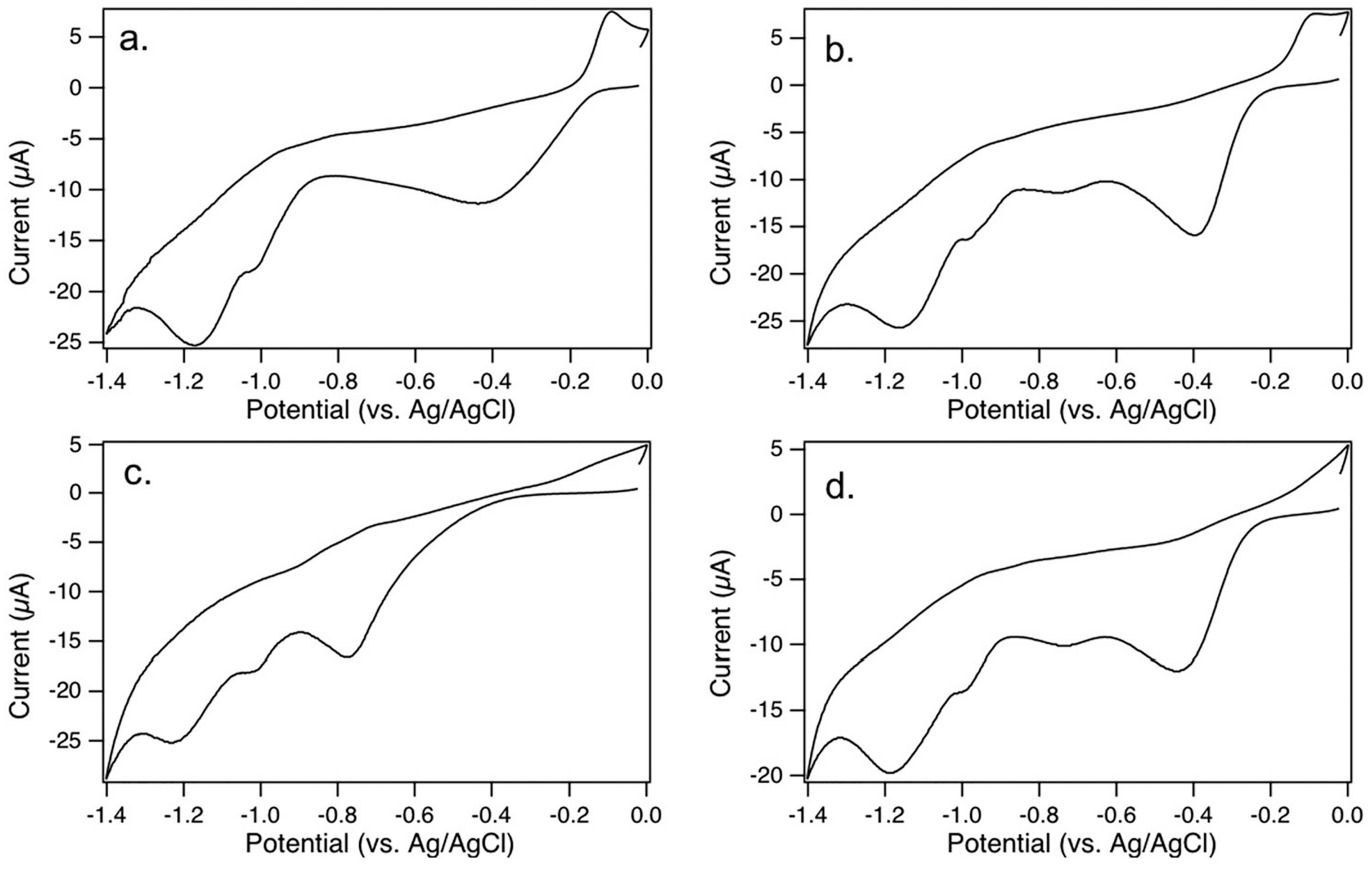
Reductive desorption cyclic voltammograms collected in 0.50 M potassium hydroxide at 0.100 V/s (a) OT; (b) 1NT; (c) 1NT/OT; (d) OT/1NT Single-component and binary SAMs were prepared with deposition conditions described earlier. Broad peaks around −0.4 are attributed to solvent desorption. Peaks attributed to SAM desorption are present between −0.7 V and −1.2 V.

We performed reductive desorption to understand the relative binding energies of the varied SAMs. Single-component molecular layers were analyzed first to understand the fundamental properties of OT and 1-NT, shown in Figure 11a and 11b, respectively. For each molecular layer there is a broad anodic response that can be attributed to oxidative readsorption.^97^ We observe several cathodic peaks for each molecular layer, which are associated with the reductive desorption process. Each molecular layer also shows a broad cathodic response around −0.4 to −0.5 V. The broad peak is the least negative peak observed and is also present in control measurements of bare gold surfaces, shown in Figure S1. We therefore attribute the broad peak to desorption of solvent species.

The reductive desorption of OT shows a prominent peak at −1.164 ± 0.010 V. We also observe a relatively small peak at −0.988 ± 0.016 V and the broad peak attributed to solvent at −0.51 ± 0.12 V. The peak at −1.164 V has a desorption potential that is consistent with previous reports of self-adsorbed molecular layers formed from linear alkanethiols on Au(111) measured in basic conditions.^79,98,99^ The relative contributions of the desorption peaks were calculated by integrating all peaks attributed to molecular desorption to determine the total desorption charge. The contribution of an individual peak was calculated as the ratio of the individual peak area to the total desorption charge. The small peak at −0.988 V contributes 4.7% of the total charge associated with the OT bound to the surface. The potential at which reductive desorption occurs can depend on a number of factors, including intermolecular interactions within the molecular layer and the crystallographic orientation of the gold surface. Reductive desorption measurements on higher-index or less thermodynamically stable gold terraces were observed to have more negative reductive desorption potentials,^82^ so the origin of the small peak at −0.988 V is more likely due to less-well-ordered portions of the molecular layer in which weaker lateral interactions are present within the molecular layer.

Reductive desorption measurements of 1NT show a more complex desorption profile. We again observe a broad peak around −0.5 V attributed to solvent background. Small peaks at more negative potentials were present at −0.742 ± 0.012 V and −0.978 ± 0.003 V, with a larger peak present at −1.155 ± 0.010 V. The large peak at −1.155 V has a potential that cannot be distinguished from the most negative desorption potential of OT found at −1.164 ± 0.010 V, which indicates similar stability of the most strongly bound components of the 1NT and OT molecular layers. The presence of two smaller peaks at less negative desorption potentials indicates the presence of bound forms of 1NT are less stable binding configurations. The presence of multiple peaks with substantial current density indicate that the 1NT molecular layers have a more heterogeneous binding configuration on the gold surface compared to OT. The presence of the less negative peaks is therefore consistent with the relatively disordered molecular layer imaged by STM and the increase in oxidation measured by XPS.

### 4.1 1NT/OT

Reductive desorption measurements of sequential deposition of 1NT followed by OT in Figure 11c reveal three peaks, with a broad peak attributed to the solvent present at a relatively low negative potential. A small peak at −0.99 ± 0.02 V is also present, and the most negative peak is present at −1.18 ± 0.03 V. The potentials of these peaks are very similar to the peaks observed for OT single-component molecular layers, with the peaks at −0.99 V and −1.18 V within the uncertainty of the corresponding peaks for OT. However, the peak at −0.99 V is substantially larger for the sequential deposition, corresponding to 9.0% of the total peak area, compared to 4.7% for the single component OT molecular layer. While we cannot distinguish the contributions of 1NT from those of OT due to the comparable reductive desorption potentials of the single-component molecular layers, the profile observed for the 1NT/OT mixed layers is consistent with the STM images showing a molecular layer in which OT has displaced 1NT but has formed a more disordered molecular ordering than a molecular layer of pure OT.

### 4.2 OT/1NT

Measurements of reductive desorption of sequential deposition of OT followed by 1NT were far less reproducible than the other surfaces measured in this work. One example is shown in Figure 11d with additional examples included in Figure S2. We observe two reproducible prominent peaks, the broad peak attributed to solvent −0.45 ± 0.06 V and a peak at −1.18 ± 0.03 V that has a desorption potential that is indistinguishable from the most negative peaks of the other three molecular layers. This peak can therefore be attributed to a relatively stable component of the molecular layer in which there are substantial intermolecular interactions. Between those peaks we typically observe either one or two additional small peaks, with potentials and sizes that lack the reproducibility observed for the single component SAMs or the 1NT/OT deposition. We attribute these peaks to molecules in less stable and more heterogeneous binding positions. STM analysis of the OT/1NT layers showed the presence of multiple surface structures, and XPS analysis of the OT/1NT molecular layers showed that the molecular layers have an oxidized component. We hypothesize that the lack of reproducibility for the less stable components of the OT/1NT molecular layers is due to variation in degree of oxidation and organization of the molecular layers.

## 5 Conclusion

Based on the observations from STM images and RD measurements, we conclude that the 1NT monolayer is highly oxidized. The appearance of the C 1s signal for 1NT at higher binding energies than 2NT and Anthracenethol is consistent with the conclusion of either submonolayer coverage or a high-defect monolayer. Due to the highly oxidized nature of the 1NT monolayer, any incoming OT molecules replace existing 1NT molecules rather than forming a stable binary monolayer composed of both compounds.

Aromatic thiols are a highly sought after family of compounds due to their potential for applications in the formation of defect-free monolayers with the capacity to participate in technologically relevant charge transfer interactions. However, there is a fundamental gap in the understanding of how the presence of a compound in a two-dimensional mixture changes the properties of the mixture components, as well as the properties of the film. To this end, it is important to map out both the variability of individual compounds and the behavior of these compounds in pristine conditions. To date, our lab has investigated three binary monolayers of aromatic and aliphatic thiol compounds at ambient conditions. The minor changes in the structural compositions of the arylthiols have led to much more significant changes in the structure of the resulting binary monolayers. These differences underline the importance of such investigations. This experiment represents the next step in the greater understanding of two-dimensional mixing as a whole. It is the goal of our research group to create a library of binary monolayers composed of aromatic and aliphatic thiols in order to identify trends in structural characteristics and deposition conditions that determine the mechanism driving the formation of binary monolayers.

## Supporting Information Available

The Supporting Information includes additional STM images, XP spectra, and CVs. Specifically: Additional STM images of bare substrate; XP spectra of single-component monolayers; Cyclic voltammograms of substrate and binary SAMs (PDF)
