# Supplementary material for: Understanding the Two-Dimensional Mixing Behavior of 1-Naphthalenethiol and Octanethiol
Source: J Phys Chem C Nanomater Interfaces. 2023 Mar 24;127(13):6531–42. doi: 10.1021/acs.jpcc.2c08822 (PMC10084448; doi:10.1021/acs.jpcc.2c08822)
Supplement: Supplementary file 1 — jp2c08822_si_001.pdf [file jp2c08822_si_001.pdf]

Supporting Information

Understanding the Two-Dimensional Mixing Behavior of 1-Naphthalenethiol and Octanethiol

Jack Sette-Ducati, Ryan Donnelly, Allison J. Molski, Emma R. Robinson, Emma K. Canning, Daniel J. Williams, Elizabeth C. Landis, L. Gaby Avila-Bront*

Department of Chemistry, College of the Holy Cross
1 College St., Worcester, MA, 01610

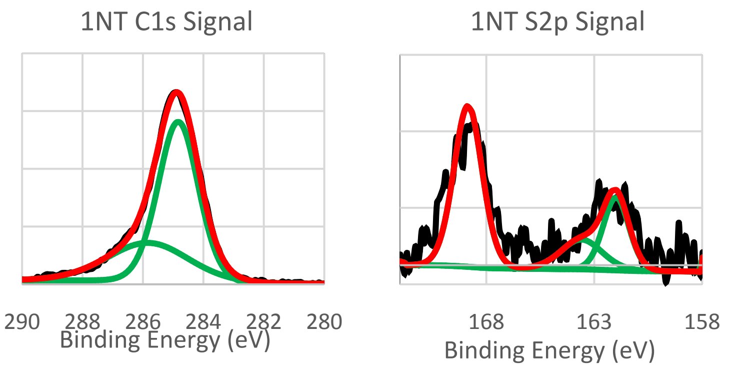

Figure S1. XP spectra of a single-component 1NT monolayer. The C1s and S2p signals are shown. Samples were prepared by immersing the gold surface into a 1 mM ethanolic 1NT solution at 60°C for 24 hours. The sample was rinsed with **ethanol** and dried under a Nitrogen stream before being analyzed with XPS. On the graphs, the raw data signal is shown in black, the curves used to fit the data are shown in green, and the final fit is shown in red.

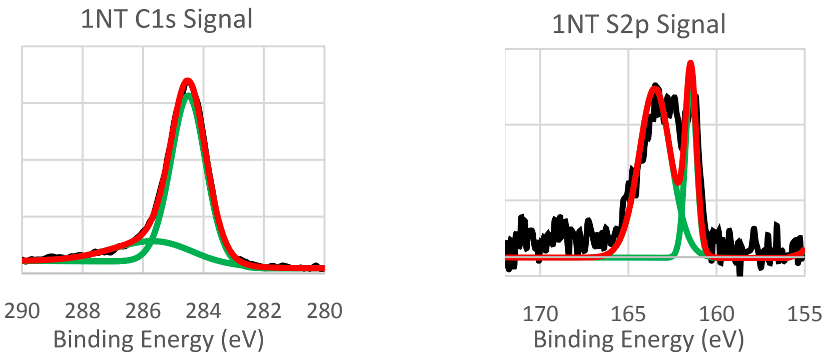

Figure S2. XP spectra of a single-component 1NT monolayer. The C1s and S2p signals are shown. Samples were prepared by immersing the gold surface into a 1 mM ethanolic 1NT solution at 60°C for 24 hours. The sample was rinsed with **benzene** and dried under a Nitrogen stream before being analyzed with XPS. On the graphs, the raw data signal is shown in black, the curves used to fit the data are shown in green, and the final fit is shown in red.

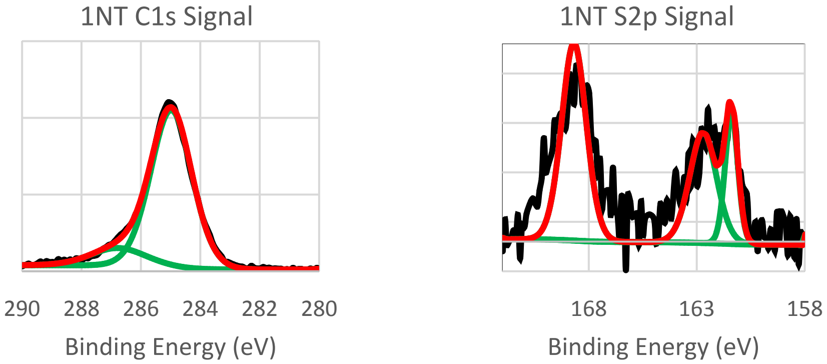

Figure S3. XP spectra of a single-component 1NT monolayer. The C1s and S2p signals are shown. Samples were prepared by immersing the gold surface into a 1 mM ethanolic 1NT solution at 60°C for 24 hours. The sample was rinsed with **toluene** and dried under a Nitrogen stream before being analyzed with XPS. On the graphs, the raw data signal is shown in black, the curves used to fit the data are shown in green, and the final fit is shown in red.

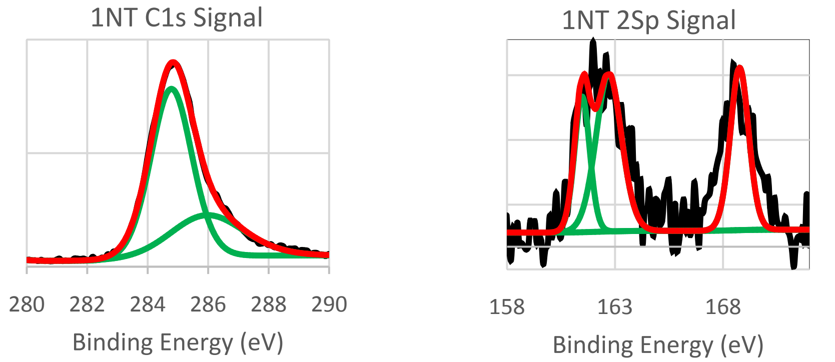

Figure S4. XP spectra of a single-component 1NT monolayer. The C1s and S2p signals are shown. Samples were prepared by exposing the gold surface to 1NT vapor at 60°C for 24 hours. The sample was rinsed with **ethanol** and dried under a Nitrogen stream before being analyzed with XPS. On the graphs, the raw data signal is shown in black, the curves used to fit the data are shown in green, and the final fit is shown in red.

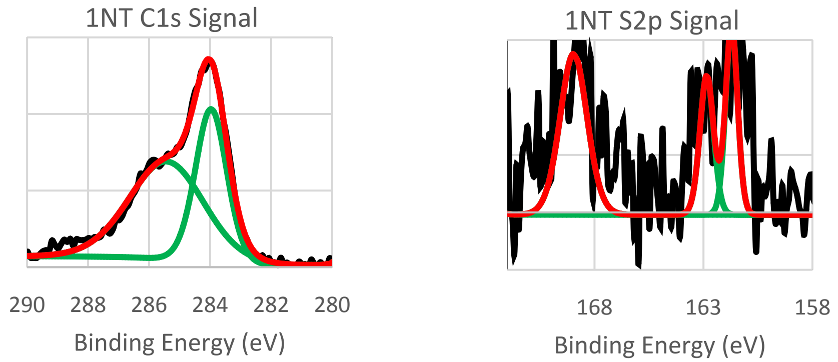

Figure S5. XP spectra of a single-component 1NT monolayer. The C1s and S2p signals are shown. Samples were prepared by exposing the gold surface to 1NT vapor at 60°C for 24 hours. The sample was **not** rinsed before being analyzed with XPS. On the graphs, the raw data signal is shown in black, the curves used to fit the data are shown in green, and the final fit is shown in red.

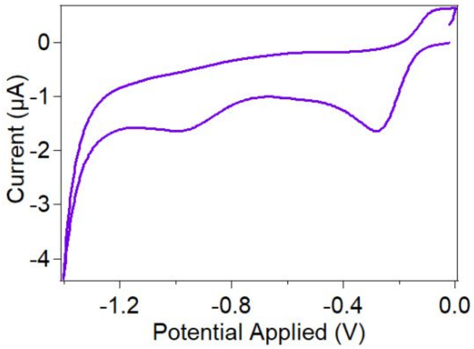

Figure S6. Reductive desorption of a bare Au electrode collected in 0.50 M potassium hydroxide at 0.100 V/s showing broad peaks due attributed to solvent.

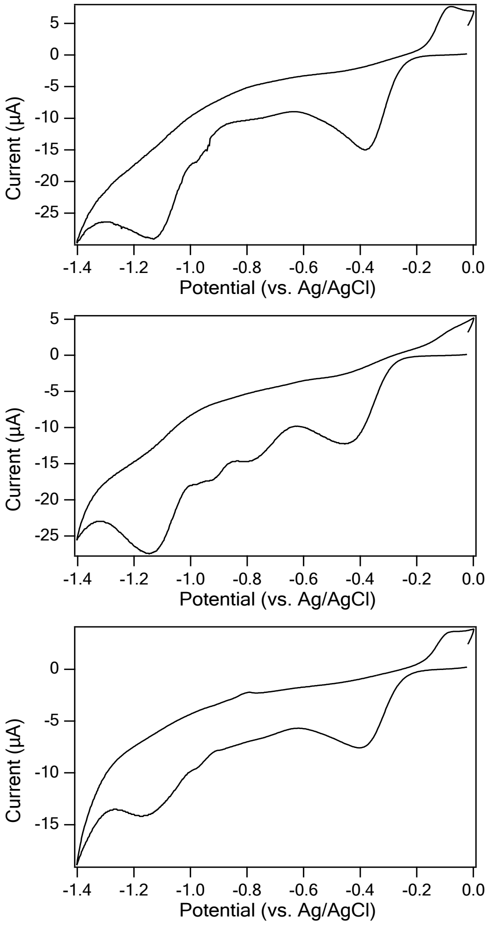

Figure S7. Reductive desorption cyclic voltammograms collected in 0.50 M potassium hydroxide at 0.100 V/s of OT/1NT binary SAMs. Samples were prepared and measured under the same conditions as Figure 11d, showing the lack of reproducibility in the desorption profiles.

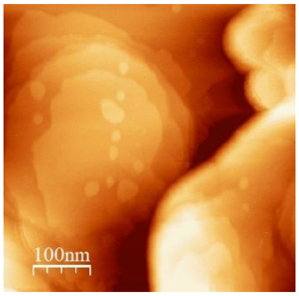

Figure S8. Representative image of bare gold sample (Au(111) on mica). This large-scale image shows several terraces as well as islands of gold on top of existing flat terrace areas. The herringbone reconstruction is not observed on these samples due to their polycrystalline nature.
